# *Sporothrix schenckii* Cell Wall Proteins-Stimulated BMDCs Are Able to Induce a Th1-Prone Cytokine Profile In Vitro

**DOI:** 10.3390/jof4030106

**Published:** 2018-09-02

**Authors:** Camila Quinello, Lucas Souza Ferreira, Isabella Picolli, Maria Luiza Loesch, Deivys Leandro Portuondo, Alexander Batista-Duharte, Iracilda Zeppone Carlos

**Affiliations:** Department of Clinical Analysis, School of Pharmaceutical Sciences, Araraquara, São Paulo State University (UNESP), Rodovia Araraquara-Jaú-Km 1, Araraquara 14800-903, SP, Brazil; caquinello@hotmail.com (C.Q.); gigabreath@hotmail.com (L.S.F.); bepicolli@yahoo.com.br (I.P.); ma_luizaloesch@hotmail.com (M.L.L.); deivysleandro@gmail.com (D.L.P.); batistaduhartea@gmail.com (A.B.-D.)

**Keywords:** *Sporothrix schenckii*, bone-marrow-derived dendritic cells, vaccine, sporotrichosis

## Abstract

Sporotrichosis is a subcutaneous mycosis affecting humans and other animals. The disease can be acquired by accidental inoculation of the fungus through the skin or through the respiratory system. Sporotrichosis can also be transmitted through bites or scratches by infected cats and more rarely by other animals (zoonotic transmission). Conventional antifungal therapy is especially inefficient in immunocompromised patients, who tend to develop the most severe forms of the disease, thus prompting the search for alternative therapies. Given their antigen-presenting properties, dendritic cells (DCs) have been used in both prophylactic and therapeutic vaccination strategies. Hence, this study aims to assess the use of DCs as a prophylactic tool in sporotrichosis by evaluating the immune profile induced by *Sporothrix schenckii* cell wall proteins (SsCWP)-stimulated, bone-marrow-derived DCs (BMDCs). Mouse BMDCs were stimulated with SsCWP for 24 h and analyzed for the surface expression of costimulatory molecules and TLR-4, as well as for the secretion of proinflammatory cytokines and IL-10. Following that, activated BMDCs were cocultured with splenocytes for 72 h and had the same cytokines measured in the supernatant. SsCWP-stimulated BMDCs showed higher expression of CD80, CD86, and CD40, but not TLR-4, and higher secretion of IL-6, IL-17A, and TNF. On the other hand, higher levels of IFN-γ, IL-10, and IL-2 were found in the supernatants of the coculture as compared with the BMDCs alone; TNF secretion was almost completely abrogated, whereas IL-6 was only partially inhibited and IL-17A was unaffected. Our results thus suggest that SsCWP-stimulated BMDCs are able to induce a Th1-prone cytokine profile which is known to be protective against other fungal diseases. This result could lead to studies which evaluate the development of prophylactic and/or therapeutic DC-based tools against sporotrichosis.

## 1. Introduction

Sporotrichosis is a fungal infection affecting humans and other animals that is caused by different *Sporothrix* species [1]. The infection can be acquired by the accidental inoculation of its causal agent through the skin or, more commonly as has occurred in Brazil in the last few years, through zoonotic transmission by infected cats with the fungus [2]. Sporotrichosis is a worldwide distributed mycosis, although it shows a higher prevalence in tropical and subtropical regions [3,4]. Over the years, many studies have attempted to develop vaccines against human- and animal-infecting opportunistic and endemic fungi [5,6,7].

It is known that several proteins located in the cell wall of *S. schenckii* are important humoral and cellular immune response inducers and are, therefore, potential candidates for diagnostic applications and as a tool in the study of vaccines to prevent sporotrichosis [8]. Pathogen-associated molecular patterns (PAMPs) have been extensively studied because of their importance in the host defense against microbes, and there has been growing recognition of the potential benefit of using PAMPs in vaccine development [9]. PAMPs deliver a “danger” signal to dendritic cells (DCs), resulting in DC activation and secretion of cytokines/chemokines, migration, maturation, antigen presentation, and costimulatory molecule expression. This, in turn, impacts B- and T-cell responses to antigens co-delivered with PAMPs [10,11].

The last decade saw a rapid progress in the comprehension of DC biology concomitantly to the development of obtaining cultures and culture methods for blood- and bone-marrow-derived DCs (BMDCs), which opened the path for DC vaccine development [12]. DCs present great potential to be used as adjuvants in both prophylactic and therapeutic vaccine formulations [13,14]. Many studies show that several immune responses are critically controlled by DCs, which are potent and widely distributed antigen-presenting cells (APCs), besides being unique in their prominent role in the activation, polarization, and regulation of adaptive immune responses [15]. DCs are highly capable in recognizing fungi-associated data and translating it into different T-cell responses both in vivo and in vitro [16]. Despite the many studies showing the use of DCs as tools for the development of vaccines against different fungi [13,17], this has been approached in sporotrichosis using complete fungal cells and exoantigens [18]. Our group reported that cell wall proteins extracted from *S. schenckii* (SsCWP) are able to induce a protective immune response against this fungi [19,20]. The present study assessed the immune response pattern induced in BMDCs when these cells were stimulated with SsCWP, as well as that produced by the cocultivation of SsCWP-stimulated BMDCs with mouse splenocytes. This is the first stage before evaluating whether stimulated DCs are able to induce a therapeutic effect in models of murine sporotrichosis.

## 2. Materials and Methods

### 2.1. Animals

Male Balb/c mice, 5–7 weeks old at the time of inoculation, were purchased from “Centro Multidisciplinar para Investigação Biológica na Área da Ciência de Animais de Laboratório” (CEMIB), Universidade de Campinas (UNICAMP), São Paulo, Brazil. Animals were housed in individually ventilated cages in an ambience with a controlled temperature and 12 h light/dark cycles. Water and food were offered ad libitum. All procedures were approved by the Institutional Ethics Committee (Protocol CEP/FCF/CAR no. 04/2014) and were in accordance with the National Institutes of Health Animal Care Guidelines.

### 2.2. Microorganism and Culture Conditions

*Sporothrix schenckii* (ATCC^®^ 16345™) isolated from a case of human lung infection (Baltimore, MD, USA) was kindly provided by the Department of Microbiology, Reference Materials Laboratory of the Oswaldo Cruz Foundation, National Institute of Quality Control in Health, Rio de Janeiro, Brazil. The fungus was kept in its filamentous phase in Mycosel™ agar at room temperature. The yeast form used in the experiments was obtained by transferring a small fragment of the mycelium into BHI (Brain Heart Infusion, Difco, Waltham, MA, USA) broth and culturing it at 37 °C under constant stirring of 150 rpm/min for 7 days. After that, 2 × 10^7^ yeast cells were transferred to a fresh medium and cultured for 5 more days in the same conditions in order to achieve a virtually 100% mycelium-to-yeast conversion in a logarithmically growing culture.

### 2.3. Extraction of the SsCWP

Extraction of the SsCWP was performed as previously described by Portuondo and colleagues (2016) with minor modifications [19]. Briefly, yeast cells collected from logarithmically growing cultures were washed with cold 25 mM Tris-HCl, pH 8.5. The yeasts were then incubated with the dithiothreitol (DTT)-based protein extraction buffer (2 mM DTT, 1 mM phenylmethyl sulfonyl fluoride, and 5 mM EDTA in Tris/HCl buffer) for 3 h at 4 °C under mild agitation. The SsCWP-containing supernatant was collected, filtered through a 0.20 µm nitrocellulose membrane (Millipore, Burlington, MA, USA), and dialyzed against distilled water at 4 °C for 48 h with water changes every 12 h and then concentrated 100 times using the Amicon^®^ vacuum system (Milipore, Burlington, MA, USA) according to the manufacturer’s instructions. The protein extract was aliquoted and stored at −20 °C, and the protein concentration was measured by the BCA assay (Pierce, Waltham, MA, USA), also according to the manufacturer’s instructions.

### 2.4. BMDCs Generation

After euthanasia, bone marrow precursor cells were extracted from the femur and the tibia of Balb/c mice and resuspended in RPMI-1640 medium (Sigma, Waltham, MA, USA) supplemented with 10% heat-inactivated fetal calf serum, 100 U/mL penicillin, 100 µg/mL streptomycin, 5 mM 2-mercaptoethanol, and 1 mM sodium pyruvate (R-10). The cell concentration was adjusted to 2 × 10^6^/mL in R-10, supplemented with 40 ng/mL murine recombinant granulocyte-macrophage colony-stimulating factor (GM-CSF) (PeproTech, Rocky Hill, NJ, USA), distributed in cultivation bottles (10 mL), and incubated in humidified air at 37 °C with 5% CO_2_. On days 3 and 6, the non-adherent cells were collected, washed and resuspended in R-10 containing 20 ng/mL GM-CSF. On day 8, non-adherent cells were collected, washed, and adjusted to 1 × 10^6^ cells/mL in R-10. An aliquot of this suspension was analyzed by flow cytometry for expression of the myeloid DC markers, CD11c and MHC-II. After that, 10^7^ BMDCs in 10 mL of R-10 were transferred to new culture bottles, stimulated or not stimulated with 50 µg/mL SsCWP for 24 h, and analyzed for the expression of CD83 and the costimulatory molecules CD86, CD80, CD40, and TLR4 by flow cytometry. The supernatant was collected and stored at −80 °C for later measurement of cytokines.

### 2.5. Flow Cytometry

The BMDCs were washed in PBS containing 1% bovine serum albumin (BSA) (Sigma), and then 1 × 10^6^ cells were stained with the following antimouse mAbs (BD Biosciences, Franklin Lakes, NJ, USA): FITC anti-CD11c (clone HL3), APC anti-I-Ab (MHC-II) (clone AF6-120.1), PE anti-CD83 (clone Michel-19), PECy7 anti-CD86 (clone GL1), PE anti-CD80 (clone 16-10A1), PE anti-CD40 (clone 3/23), and PECy7 anti-CD284 (TLR4) (clone SA15-21, BioLegend, San Diego, CA, USA). The cells were acquired using a BD Accuri C6 flow cytometer (BD Biosciences) and 20,000 events of the total cells by using an FSC-H (forward scatter-height) gate as a parameter for the exclusion of cellular debris. The analysis was conducted using BD CSampler Software, and the BMDCs were identified by the expression of CD11c and MHCII from all cells obtained, using cells not marked as a parameter for the correct positioning of the gates. Cell activation markers were analyzed within the CD11c^+^MHCII^+^ population, both stimulated and nonstimulated with SsCWP, and the results were presented as the Median Fluorescence Intensity (MFI).

### 2.6. Splenocytes

After euthanasia, spleens were aseptically removed and passed through a 100 µm cell strainer into a petri dish containing 2 mL of PBS with the aid of a syringe plunger. For red cell lysis, the resulting suspension was added with 6 mL of a 0.17 M ammonium chloride solution and then incubated on ice for 5 min. The splenocytes were then separated from the supernatant by centrifugation at 300× *g* for 5 min at 4 °C, washed once with 3 mL of R-10 medium, resuspended in 1 mL of the same medium, counted using the Trypan blue exclusion test, and then adjusted to the desired concentration.

### 2.7. Cocultivation of BMDCs and Splenocytes

To assess the inducing properties of the SsCWP-stimulated BMDCs, activated BMDCs that were previously washed and adjusted to 10^5^/mL were cocultured with splenocytes in a 1:5 or 1:10 ratio (BMDC: splenocytes) in a 96-well plate for 72 h at 37 °C with 5% CO_2_. Splenocytes or SsCWP-stimulated BMDCs alone were used as controls. The plates were then centrifuged at 300× *g* for 5 min at 4 °C and the supernatants were kept at −80 °C until cytokine determination.

### 2.8. Cytokine Measurement

Cytokines were measured using the mouse Th1/Th2/Th17 cytokine cytometric bead array (CBA) kit (BD Biosciences), according to the manufacturer’s instructions using a FACS LRS II Fortessa flow cytometer (BD Biosciences). The data were analyzed using FlowJo (Tree Star, Ashland, OR, USA), and the results of cytokine concentration were expressed in pg/mL [21].

### 2.9. Statistical Analysis

Statistical analysis was performed in GraphPad Prism ver. 6.01 by using a one-way analysis of variance (ANOVA) with Tukey multiple comparisons test. *p <* 0.05 was considered to be statistically significant.

## 3. Results

### 3.1. Activation and Cytokine Profile of SsCWP-Stimulated BMDCs

**Figure 1:**
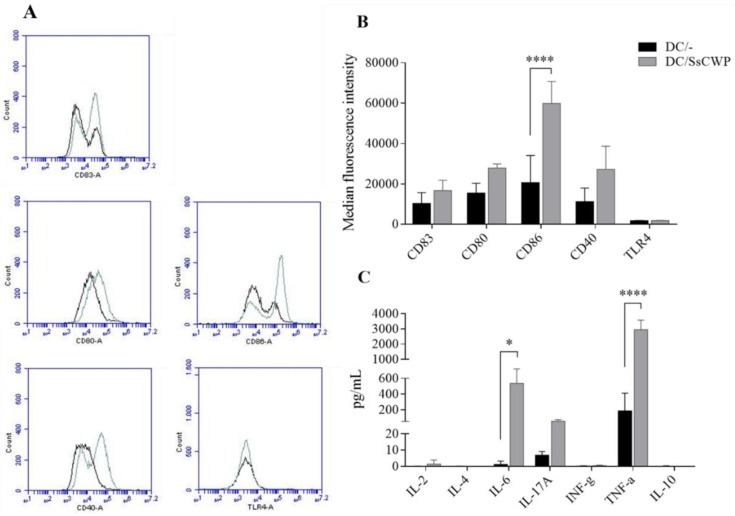
Phenotypical analysis of stimulated bone-marrow-derived dendritic cells (BMDCs). (**A**) Histograms representative of expression analysis of CD80, CD86, CD40, and TLR-4 (cellular activation markers) and CD83 (maturation marker) in BMDCs stimulated or not with SsCWP. (**B**) Cell surface expression of indicated markers on BMDCs. (**C**) Cytokine concentration in the culture supernatant of BMDCs. The data are presented as the mean ± SD of 3 three culture of pooled cells obtained from bone marrow of mice and the expression of markers are presented as their median fluorescence intensity (MFI). DC/-: unstimulated BMDCs (black line); DC/SsCWP: SsCWP-stimulated BMDCs (grey line). Asterisk indicates statistically significant difference between unstimulated and SsCWP-stimulated BMDCs: * *(p* < 0.05) and **** (*p* < 0.0001) as indicated. DC: dendritic cell. SsCWP: *Sporothrix schenckii* cell wall protein.

The protocol used for BMDC generation resulted in 85.5 and 89.0% of CD11c^+^MHCII^+^ cells with the average of 3 cultures of BMDCs, before and after stimulation with SsCWP, respectively. The phenotypical analysis of BMDCs revealed a higher expression of the maturation marker CD83, as well as the costimulating molecules CD80, CD86, and CD40, but not TLR4, after 24 h (Figure 1A,B). Measurement of the cytokines released in the supernatants of the BMDC cultures revealed higher levels of IL-6, IL-17A, and TNF after stimulation with the SsCWP (Figure 1C).

### 3.2. Cytokine Profile Induced by BMDCs Upon Cocultivation with Splenocytes

**Figure 2:**
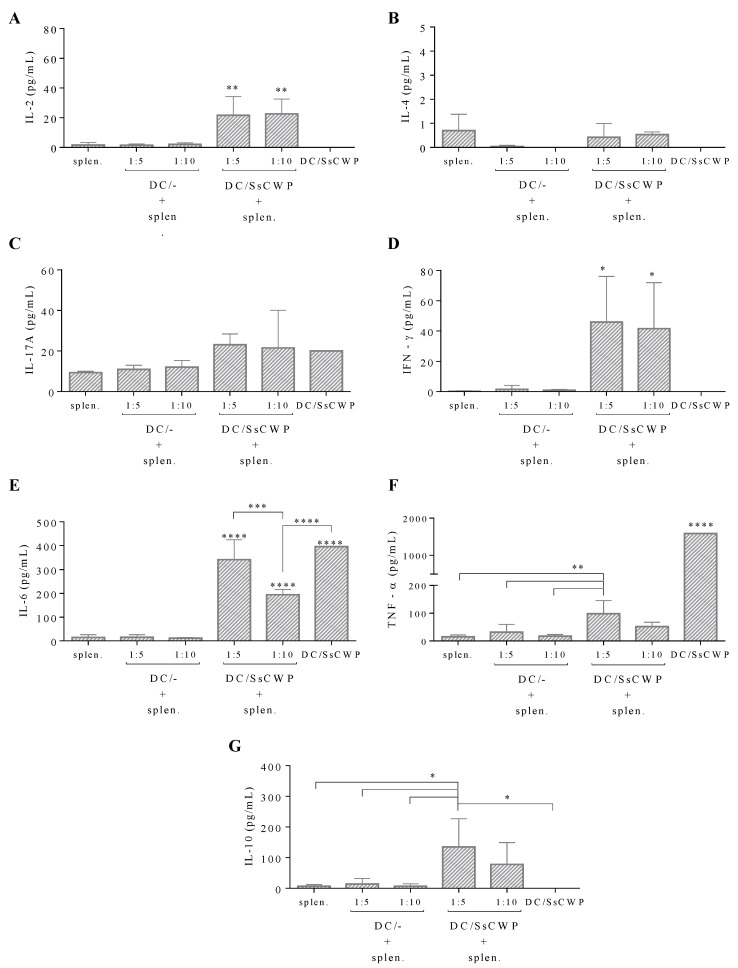
Measurement of cytokines released in the BMDC: splenocyte coculture supernatant. Values are presented as the mean ± SD of four animals. Asterisk indicates statistically significant difference between groups: * *(p* < 0.05), ** (*p* < 0.01), *** (*p* < 0.001), and **** (*p* < 0.0001) as indicated.

To assess the value of SsCWP-stimulated BMDCs as a vaccine adjuvant, the ability of these cells to activate T cells was tested in vitro by cocultivation with total splenocytes for 72 h and then measuring the cytokines released in the coculture supernatant. Our results show that in both 1:5 and 1:10 (DC: splenocyte) ratios, SsCWP-stimulated BMDCs were able to induce a significant increase in the release of IL-2 (Figure 2A) and IFN-γ (Figure 2D) but not IL-4 (Figure 2B) or IL-17A (Figure 2C) as compared with unstimulated BMDCs; IL-10 was only significantly induced in the 1:5 ratio (Figure 2G). As before, BMDCs released relatively high TNF amounts when cultured alone, but showed an unexpected, almost complete inhibition of this cytokine upon cocultivation with splenocytes (Figure 2F). Conversely, IL-6 levels were mostly maintained in the coculture, although significantly decreasing in the 1:10 ratio as compared with SsCWP-stimulated BMDCs alone or with the 1:5 ratio (Figure 2E).

## 4. Discussion

The use of DCs in vaccinations is promising as they lie in the intersection between the innate and the adaptive immunity, uniquely being able to capture and process antigens for presentation to T cells through MHC class II molecules. DCs recognize fungi through a wide range of pattern recognition receptors (PRRs) such as toll-like receptors (TLRs), which are located both extracellularly and intracellularly. Fungi recognition results in cytokine release and surface expression of the costimulating molecules CD80, CD86, and CD40 by DCs, which are necessary to direct the differentiation of naive CD4^+^ T cells into a T helper phenotype [22]. As it is already known, the activation and expansion of T cells and their acquisition of effector functions are key to the development of adaptive immune responses, with a substantial part of T cell proliferation and differentiation being traced back to the initial encounter of DCs with a given antigen [23]. For decades, a variety of cell wall proteins from many different pathogenic fungi have been evaluated in mouse models of vaccinations for their immunogenicity, safety, and protection-affording potential [6]. Although very few clinical trials have been performed in humans, a growing number of antifungal vaccine candidates are being evaluated in preclinical studies as part of the renewed interest in the potential use of vaccines as replacements or adjuncts to chemotherapy to reduce antifungal drug use and consequently limit drug resistance and toxicity [7]. In the study presented here, we observed that the SsCWP are able to promote BMDCs maturation as well as their activation, as shown by the increased expression of CD83 and CD80, and CD86 and CD40 post-stimulation, respectively. This leads us to believe that it is possible to use DCs as a vaccine for sporotrichosis. Other studies using fungal antigens as DC activators showed their capacity to promote the maturation of these cells, as shown by the increased expression of MHC class II and costimulatory molecules involved in antigen presentation and T cell activation [24,25]. However, it is known that fungal wall proteins are associated with carbohydrates forming glycoprotein complexes such as mannans and glycans, which can directly activate dendritic cells and macrophages through their interaction with innate immune receptors such as C-type lectin receptors and toll-like receptors. These activation pathways have already been described in sporotrichosis [26,27]. A deeper characterization of the polysaccharide components associated with the isolated proteins is necessary to identify with more precision the possible activation pathways of BMDCs observed in this study. 

Regarding cytokine production by the SsCWP-stimulated BMDCs, our data showed an increased production of IL-6, IL-17A, and TNF, suggesting that SsCWP-stimulated BMDCs could induce, in vivo, a Th17 pattern inflammatory response. Contrary to our expectations, however, these BMDCs induced a predominantly Th1 cytokine profile, as noted by increased IL-2 and IFN-γ and only basal levels of IL-17A and IL-4 when cocultured with splenocytes. It has been previously indicated that granuloma formation in sporotrichosis may be associated with a Th1 response in the skin lesions, as evidenced by the local detection of IFN-γ as well as the fact that *S. schenckii* of cutaneous origin is a more potent inducer of Th1-prone DC activation than that of visceral origin [28]. Traditionally, responses mediated by IFN-γ-producing Th1 cells are considered to be responsible for conferring protection against fungi, while IL-4-mediated Th2 responses lead to an increased susceptibility [29]. However, the inflammatory response in sporotrichosis also has an important neutrophilic component, as evidenced by the influence of the Th17-mediated inflammation [30,31]. Furthermore, a previous study performed in our lab showed that *S. schenckii*-primed BMDCs were able to promote a Th1- and Th17-biased response when cocultured with splenocytes extracted from mice that had been previously challenged intraperitoneally with *S. schenckii*, as shown by increased IFN-γ and decreased IL-17A release [18]. Other studies reported that DCs are able to phagocytose *S. schenckii* and to induce a Th1-prone cytokine profile, as well as to induce the proliferation of T lymphocytes that had been presensitized with *S. schenckii* [32]. Lastly, TNF release was abrogated whereas IL-10 was induced when SsCWP-stimulated BMDCs were cocultivated with splenocytes. As IL-10 can directly inhibit IL-2, TNF-α, and IL-5 production [33], it seems that this pathway could be responsible, at least partially, for this finding.

Although very few clinical trials have been performed in humans, a growing number of antifungal vaccine candidates are being evaluated in preclinical studies as part of the renewed interest in the potential use of vaccines as a replacement or adjunct to chemotherapy to reduce the use of antifungal drugs and consequently limit drug resistance and toxicity [7]. Moreover, one of the most interesting aspects is to achieve an adequate efficacy/toxicity balance of the vaccines as adjuvants frequently cause adverse reactions [34]. For this reason, the search for alternatives using molecular modulators seems to be a promising way [35]. Since activated DCs may be more efficient than nonspecific commercial adjuvants, we propose that SsCWP-stimulated BMDCs could represent a potential therapeutic tool for sporotrichosis management. New studies of immunogenicity and protection are needed to confirm the true usefulness of this proposal.

## 5. Conclusions

In summary, our results indicate that SsCWP are able to activate BMDCs to acquire an activated phenotype that promote a Th1 bias, which leads us to believe that a SsCWP-stimulated, BMDCs-based vaccine could be evaluated as a potential tool for sporotrichosis immunotherapy.
